# Effects of the COVID-19 Pandemic on Everyday Neurosurgical Practice in Alsace, France: Lessons Learned, Current Perspectives, and Future Challenges—Preliminary Results of a Longitudinal Multicentric Study Registry

**DOI:** 10.3390/medicina60030390

**Published:** 2024-02-25

**Authors:** Guillaume Dannhoff, Charles-Henry Mallereau, Mario Ganau, Biagio Roberto Carangelo, Giorgio Spatola, Julien Todeschi, Lara Prisco, Rodolfo Maduri, Marie des Neiges Santin, Sandrine Woelffel, Isabella Mastrobuono, Jimmy Voirin, Franco Moruzzi, Beniamino Nannavecchia, Vitaliano Francesco Muzii, Alessandro Zalaffi, Carmen Bruno, Salvatore Chibbaro

**Affiliations:** 1 Neurosurgery Department, Strasbourg University Hospital, 67000 Strasbourg, Francemario.ganau@alumni.harvard.edu (M.G.); giorgio.spatola@univ-amu.fr (G.S.); mariedesneiges.santin@chru-strasbourg.fr (M.d.N.S.); 2 Neurosurgery Unit of “Dipartimento di Scienze Mediche Chirurgiche e Neuroscienze”, Siena University Hospital, 53100 Siena, Italy; 3 Swiss Medical Network, Clinique de Genolier, 1272 Genolier, Switzerland; maduri@hin.ch; 4 Icademie School, 83000 Toulon, France; 5 Assistance and Chronicity Unit of Alto Adige NHS Trust, 39100 Bolzano, Italy; 6 Neurosurgery Department, Pasteur Hospital, 68000 Colmar, France; 7 Neurosurgery Department, Ospedale G. Mazzini Teramo, 64100 Teramo, Italy; beniamino.nanavecchia@chru-strasbourg.fr; 8 Neurosurgery Department, Andria Hospital, 76123 Andria, Italy

**Keywords:** COVID-19, neurosurgery, SARS-CoV-2 pandemic, emergency, coronavirus

## Abstract

*Background and Objectives*: The global outbreak caused by the SARS-CoV-2 pandemic disrupted healthcare worldwide, impacting the organization of intensive care units and surgical care units. This study aimed to document the daily neurosurgical activity in Alsace, France, one of the European epicenters of the pandemic, and provide evidence of the adaptive strategies deployed during such a critical time for healthcare services. *Materials and Methods*: The multicentric longitudinal study was based on a prospective cohort of patients requiring neurosurgical care in the Neurosurgical Departments of Alsace, France, between March 2020 and March 2022. Surgical activity was compared with pre-pandemic performances through data obtained from electronic patient records. *Results*: A total of 3842 patients benefited from care in a neurosurgical unit during the period of interest; 2352 of them underwent surgeries with a wide range of pathologies treated. Surgeries were initially limited to neurosurgical emergencies only, then urgent cases were slowly reinstated; however, a significant drop in surgical volume and case mix was noticed during lockdown (March–May 2020). The crisis continued to impact surgical activity until March 2022; functional procedures were postponed, though some spine surgeries could progressively be performed starting in October 2021. Various social factors, such as increased alcohol consumption during the pandemic, influenced the severity of traumatic pathologies. The progressive return to the usual profile of surgical activity was characterized by a rebound of oncological interventions. Deferrable procedures for elective spinal and functional pathologies were the most affected, with unexpected medical and social impacts. *Conclusions*: The task shifting and task sharing approaches implemented during the first wave of the pandemic supported the reorganization of neurosurgical care in its aftermath and enabled the safe and timely execution of a broad spectrum of surgeries. Despite the substantial disruption to routine practices, marked by a significant reduction in elective surgical volumes, comprehensive records demonstrate the successful management of the full range of neurosurgical pathologies. This underscores the efficacy of adaptive strategies in navigating the challenges imposed by the largest healthcare crisis in recent history. Those lessons will continue to provide valuable insights and guidance for health and care managers to prepare for future unpredictable scenarios.

## 1. Introduction

The COVID-19 pandemic, triggered by the emergence of the novel SARS-CoV-2 virus, has shocked the world ever since late 2019, including various waves throughout 2020 and 2021. Those events were perceived across countries and continents and served as reminders of the intrinsic vulnerability of our society and the need for collective action in cases of global crisis [1,2,3]. In 2020, COVID-19 heavily affected the European Union, where the mortality rate increased by 11% and interrupted the paradigm of an ever-increasing life expectancy that had constantly characterized the prior five decades. As of 3 January 2023, 665,740,989 cases of SARS-CoV-2 infections were officially recorded worldwide, resulting in 6,699,947 deaths. France was particularly affected, with 39,333,268 cases and 162,214 deaths reported among its population of 65,584,518 [4]. 

This unprecedented situation forced the medical community to not only issue guidelines and modify clinical pathways but also to radically rethink the organization of healthcare systems and address the needs of entire populations. This phenomenon required consideration for better triaging, prioritization, and rationing of accessible resources [1]. From a neurosurgical perspective, the high virulence of SARS-CoV-2 and the severe natural history of COVID-19 eroded the availability of high dependency unit (HDU) and intensive care unit (ICU) beds. Furthermore, the lockdown in many countries necessitated the immediate modification of working routines, resulting in a dramatic decrease in elective surgeries and, in some cases, a shutdown of elective neurosurgical services [2,5,6,7,8,9,10,11,12].

Those measures required governments and healthcare organizations to accept a departure from well-established and globally accepted standards of care. In critical situations, neurosurgeons encountered the difficulties of allocating scarce healthcare resources, leading to increased mortality or significantly less favorable outcomes, particularly in oncology patients [13,14,15]. Many countries and international scientific societies rapidly developed and approved new recommendations, best practices, and checklists for immediate implementation [16,17,18,19,20,21]. The northeastern part of France witnessed a notably high number of recorded cases, emerging as one of the pandemic’s epicenters in the country and throughout continental Europe. With specific regards to decision-making in neurosurgery, the rationale was always based on the need to address rapid clinical decompensation attributed to elevated intracranial pressure or evolving neurological deficits [19,22]. 

Alsace notably includes two large hospitals with a neurosurgery department: the regional University Hospital of Strasbourg and a non-teaching hospital in Colmar, both operating on a network model coordinated by a local interconnected crisis unit. With a total population of 2 million inhabitants, these two neurosurgery departments employ 18 board-certified neurosurgeons and 10 trainees [3,23]. In the initial stages of the pandemic, policymakers and administrators were compelled to reallocate resources toward frontline healthcare professionals and reduce non-urgent procedures, prioritizing the emergency care of patients with severe pneumonia, a growing number of whom tested positive for SARS-CoV-2 [24]. Initially, the most impactful measure for slowing the infection in communities was social distancing. However, the healthcare sector had to adjust to the surge in infections by reallocating human and technological resources from various specialties to address the strain on overwhelmed ICUs. The Alsace hospital crisis authority initially made the decision to release all frontline medical personnel (emergency departments, ICUs, infectious diseases departments, etc.) aged over 65 years or those over 60 years with relevant co-morbidities. Subsequently, a call for volunteers was issued, and additional personnel were deployed to the frontline or, in the case of retired specialists without comorbidities, assigned to tasks "behind the lines," encompassing vital logistical activities such as planning, organizing, monitoring services, conducting telemedicine consultations, and coordinating call centers across the region. It quickly became evident that this unforeseen pandemic would significantly disrupt not only neurosurgical practices but also related specialties, including ENT, ortho-spine, oncology, radiation therapy, etc.

Starting from 15 March 2020, every department within these hospitals swiftly restructured on-call schedules, implemented new triaging protocols to identify patients in need of immediate neurosurgical intervention, and halted elective procedures to make ventilators and anesthetists available. These measures mirrored actions taken in various other countries, including China and Italy [21,25,26,27,28]; nevertheless, our guiding principles and specific internal and external initiatives contributed to the distinctive characteristics of our crisis response. We assert that these actions warrant documentation to enrich the body of literature on preparedness. This documentation may serve not only in preparation for future epidemic outbreaks but also for any significant crisis disrupting the routine provision of healthcare services.

In this context, the authors aim to convey their experience in the Alsace region of France, elucidating the significant and sudden transformation of neurosurgical work patterns to ensure the ability to continuously serve the needs of an entire population. This study will describe the development of effective management strategies and explore the findings of a prospective longitudinal multicenter study.

## 2. Materials and Methods

### 2.1. Study Design

A multicenter observational prospective registry was held, gathering data on a cohort of 3842 patients managed in Alsace during a 24-month period (from 15 March 2020 to 15 March 2022). Some data can be compared with historical clinical information obtained from electronic patient records (EPRs) for clinical provision in previous years. 

### 2.2. Ethics

This investigation adhered to the Ethical Principles for Medical Research Involving Human Subjects outlined in the 2004 Declaration of Helsinki, with subsequent revisions in 2008 and 2013. Ethical clearance and registration for clinical trials were secured from the local Institutional Review Board (IRB) under study number CE-2020-71 on 30 April 2020. Additionally, the COVID-19 Ethical Committee also reviewed and endorsed this study protocol. The reporting of the study results is in keeping with the guidelines of the Strengthening the Reporting of Observational Studies in Epidemiology (STROBE) statement for observational studies and checklist [29].

### 2.3. Objectives

This study sought to provide a longitudinal description of neurosurgical activities in Alsace, capturing the impact of five subsequent waves of the COVID-19 pandemic. The prospective database facilitated the primary objective: depicting the “daily neurosurgical activity” conducted during the lockdown stage from March to May 2020, along with its temporal evolution up to March 2022. The secondary objective was to evaluate performance during the COVID-19 period in comparison to routine circumstances (non-restrained surgical activity conducted during pre-pandemic conditions in March–May 2019). Finally, the tertiary objective was to analyze the long-term side effects of the pandemic on neurosurgical patients/diseases, elaborating on future management strategies and possible challenges.

### 2.4. Criteria

The departmental performance was evaluated based on the availability of ICU beds and ventilators, key criteria for gauging the severity and impact of the pandemic. All objectives involved a meticulous assessment of the key pathologies and associated surgical procedures, considering their urgency and surgical outcomes. Throughout this period, the study’s primary interests included the mechanisms of traumatic injuries, the oncologic profile and management of malignant tumors, and the characteristics of subarachnoid hemorrhage.

### 2.5. Standard Local Setup, Reorganization, and Evolution Up-on-Time

The neurosurgical units at Strasbourg University and Colmar Hospitals are collectively composed of 80 regular beds and 10 high dependency care unit (HDU) beds. The annual average number of surgeries across both centers is approximately 4000, serving a population of around 2,000,000 individuals and encompassing various neurosurgical domains such as functional, vascular, neuro-oncology, hydrocephalus, degenerative spine, brain and spine trauma, pediatric, and epilepsy surgery. The established standard for ICU beds is 140, with an average bed occupancy rate of 85% (of note, in 2012, there were 210 ICU beds in Alsace, with the number progressively lowering to 172 in 2016 and 140 in 2019). On 29 March 2020, the Regional Health Agency (ARS) announced that, in just a few weeks, the capacity of intensive care beds had been multiplied by a factor of 3, reaching a total of 420. Such an increase had been achieved by reorganizing the existing capabilities without adding any new temporary structure.

The standard capacity of the neurosurgical operating theaters in these 2 main regional hospitals is as follows: 5 operating theaters per day, from 8 am to 5 pm, from Monday to Friday. During the lockdown period, capacity was reduced to 2 operating theaters (one for each hospital) once a week for elective procedures. Another extra theater was dedicated to very selected and extremely emergent cases to be shared by all surgical and interventional specialties on each site.

The goal of such reorganization was to redirect and distribute all the operating staff, especially the scrub and anesthesiology nurses, to the newly created ICU and HDU beds. For example, in the period March–May 2020 at the Hautepierre hospital where the neurosurgical unit is located, only 3 of 32 theaters were available per day: a stand-alone operating theater assigned to urgent COVID-19-confirmed patients, an operating theater for urgent non-COVID-19 patients, both operating around the clock, and a third operating theater available for 48-h-deferrable patients, which was utilized collectively by all seven surgical specialties within the hospital. 

The above organization evolved over time as follows:

(a) From 1 June 2020 until 28 February 2021, the availability of surgical theaters increased to 2 per week for each neurosurgical center.
(b) From 1 March 2021 until 31 October 2021, the availability of surgical theaters increased to 3 per week for each neurosurgical center.
(c) From 1 November 2021 until 15 March 2022, the availability of surgical theaters increased to 4 per week for each neurosurgical center.
(d) From 16 March 2022, up to date, the availability of surgical theaters increased to the pre-pandemic normal standard activity level, with a total capability of 25 operating theaters per week for the 2 neurosurgical centers.

### 2.6. Local Epidemiology Context

The southern region of Alsace, encompassing the Haut-Rhin department with major cities like Mulhouse and Colmar, emerged as a significant cluster of COVID-19 cases around mid-February 2020. By the end of the month, the northern region, including the Bas-Rhin department with the main city of Strasbourg, experienced a similar pattern of widespread transmission. During the peak of the outbreak (March–May 2020), critical COVID-19 patients from all three cities had to be transferred to other French hospitals and even abroad due to the saturation of internal resources. A nationwide state of health confinement was declared from 17 March to 11 May 2020.

### 2.7. Patients

All patients admitted to the neurosurgical departments amid the SARS-CoV-2 pandemic, along with neurosurgical referrals and external consultations, were included in a prospective registry covering the two-year period from 15 March 2020 to 15 March 2022. The registry encompassed every adult patient in need of neurosurgical care within the regional network; pediatric patients admitted to the local pediatric surgery department were not included in this study.

### 2.8. Data Collection

Anonymized hospital identification number, birthdate, gender, cause of admission, clinical symptoms, COVID-19 status, surgical procedures (if conducted), and their level of urgency (refer to Table 1) were recorded. Priority criteria for surgeries were determined based on a previously outlined grading system [30], and triaging categories included emergency (EM group) for those needing immediate surgical intervention within hours, deferrable (UP group) for patients requiring treatment within 7–15 days, and elective (EL group) for those needing treatment within two months. A positive COVID-19 status was defined as a positive SARS-CoV-2 PCR on a nasopharyngeal swab and/or the presence of characteristic radiographic lesions on a chest CT scan [25,28].

### 2.9. Endpoints

To assess the severity of the COVID-19 pandemic and the coping strategies conceived and adopted during this period, the following parameters were examined: (a) the total count of COVID-19-positive patients admitted to the ICU for mechanical ventilation; (b) COVID-19-positive patients in need of neurosurgical care; (c) COVID-19-positive patients transferred to external hospitals; and (d) the number of patients relocated to another medical center due to the unavailability of optimal surgical management. Neurosurgical care was characterized by either the execution of a surgical procedure by a neurosurgeon or medical attention and conservative treatment within a neurosurgical unit.

Patients were categorized into distinct classes, encompassing traumatic, neurovascular, infectious, neuro-oncology, hydrocephalus, degenerative spine, and functional neurosurgery. Pertinent parameters specific to each pathological condition were duly documented, including the mechanism of trauma, the presence of any intoxication (alcohol, drug addiction, etc.), and the Glasgow Coma Scale (GCS) [31] for traumas. For subarachnoid hemorrhage cases, the World Federation of Neurosurgical Societies (WFNS) score [32] was noted, while neuro-oncology cases included the Karnofsky Prognostic Scale (KPS) and the therapeutic strategy (biopsy/surgical removal). These endpoints were compared with analogous data available from a matching cohort selected from the institutional pre-pandemic historical database.

### 2.10. Statistical Analysis

Variables were categorized as either continuous or categorical. Descriptive statistics, including mean, range, and median, were employed for continuous data. Categorical data were expressed as total counts and proportions. Fisher’s exact test was applied to assess associations within the studied variable. A *p*-value < 0.05 was deemed statistically significant. The statistical analysis of the study was conducted using the GraphPad online calculator 10 (http://www.graphpad.com/quickcalcs/, accessed on 2 April 2020).

## 3. Results

### 3.1. Local Impact of the SARS-CoV-2 Pandemic (Focus on the Strasbourg Hautepierre University Hospital)

The severity and intensity of the COVID-19 pandemic can be illustrated by ICU bed saturation [23]. At the Strasbourg Hautepierre University Hospital, the average ICU bed capacity peaked at 213% on 2nd April 2020, and remained over 163% for 57 consecutive days. On average, there were 5 COVID-19-positive patients for every one patient needing mechanical ventilation for another condition. This ratio climbed as high as 20 to 1. Such a peak during the lockdown period required the transfer of 56 COVID-19-positive patients to other regional healthcare facilities, including those abroad in Germany and Switzerland.

### 3.2. Neurosurgical Care during Lockdown

In Strasbourg University Hospital (Hautepierre) and in Colmar Regional Hospital, a total of 325 patients received neurosurgical care (i.e., surgery or medical care under neurosurgeons’ supervision). A total of 189 (58%) of them underwent a surgical or neurointerventional procedure (Table 2). Within the surgical patient cohort, urgency levels were distributed as follows: 60 emergency (EM) patients, encompassing 31.7% of the surgical group; 96 urgent (UP) patients, representing 50.8%; and 33 elective (EL) patients, accounting for 17.5%.

Among the 325 patients, only 4 individuals (1.23%) needed to be transferred to a different neurosurgical facility. These transfers were necessary due to the following conditions: 2 cases of brain metastasis (one from melanoma and one from breast cancer, both requiring awake surgery), as well as 2 cases of lumbar herniated discs leading to cauda equina syndrome. A total of 29 individuals (8.92%) tested positive for COVID-19 initially, and an additional 5 patients, initially negative, converted to positive during their in-hospital stay. The durations for these status conversions were 22, 14, 11, and 8 days, respectively.

**Figure 1:**
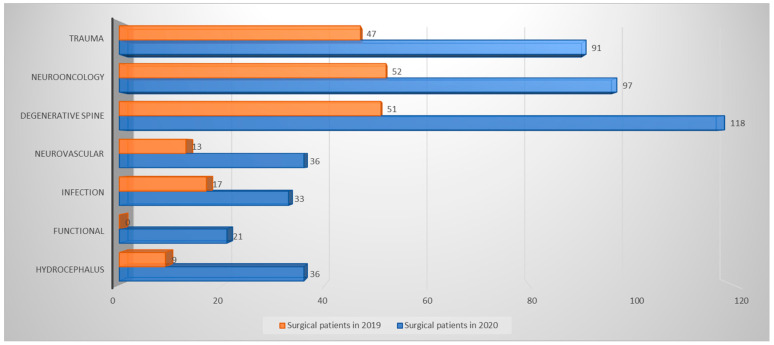
Diagram comparing the neurosurgical activity performed during lockdown in 2019 (orange bars) and during the corresponding period in 2020 (blue bars). The number of surgeries performed by pathologies is detailed next to each bar.

Comparing the same period in 2019 and 2020 across the 2 hospitals (Figure 1), there was certainly a significant decline in the number of surgeries performed. In 2019, a total of 432 surgeries were conducted, while only 189 surgeries took place in 2020. Notably, functional procedures and calvarial reconstruction, both necessitating the insertion of standardized or patient-specific implants, were systematically postponed during the lockdown period. Otherwise, the pathologies treated remained similar between these two periods. 

### 3.3. Relevant Pathologies Impacted

Among the main variations in pathologies encountered during lockdown, the following elements are to be noted:

(a) Most traumatic brain injuries (TBI) were mild, as only 3 out of 47 were severe (initial GCS < 8/15). Notably, most elicited traumas stemmed from low-speed impacts, with excessive alcohol consumption identified as the primary cause of injury in 32 cases.
(b) Other decompensated chronic conditions, such as diabetic polyneuropathy, Parkinson’s disease, and Alzheimer’s disease, were associated with 13 cases.
(c) Among neuro-oncological cases, it is noteworthy that no glioblastoma was surgically removed. Surgery did not appear adequate in any of the 16 cases, either due to the poor general condition and/or multifocal extension at diagnosis. All cases thus benefited from a stereotactic biopsy alone. This is a significant statistical difference from the care provided the previous year (*p*-value < 0.011), as glioblastomas treated in the same period in 2019 were mostly surgically resected (14 out of 21 cases), whereas the remaining 7 cases (30%) underwent biopsy alone.
(d) Only 3 patients, all of whom presented with pituitary apoplexy accompanied by a sudden loss of visual acuity, underwent endoscopic endonasal procedures. The surgeries were meticulously performed, incorporating all the precautions outlined by skull base surgery societies [1,2,3].
(e) Within the 7 patients who were admitted for an aneurysmal subarachnoid hemorrhage (aSAH), 1 presented as severe at diagnosis (WFNS grade 5) and died shortly after admission. The remaining patients exhibited a good prognosis at diagnosis (4 WFNS grade 1 and 2 WFNS grade 2) and could benefit from surgical clipping (4 patients) or endovascular treatment (2 patients). It is worth noting that between 15 March and 12 May 2019, 9 cases of aSAH necessitated treatment.

### 3.4. Results/Analysis of Neurosurgical Activity from 1 June 2020 until 28 February 2021 (Alsace Region)

In this second period where the availability of operating theaters doubled from 1 to 2 a week in each hospital, the global neurosurgical activity regarding the case mix remained very similar to the previous activity with non-significant differences (Table 3); the only exception was regarding functional procedures (deep brain stimulation (DBS), epilepsy and pain surgery, and spastic rigidity managed by intrathecal baclofen) that were further systematically postponed, causing a heavy medical and ethical issue that will be discussed in more detail in the discussion section. From March 2020 to March 2022, 90 patients affected by Parkinson’s disease, 53 affected by resistant and medically intractable epilepsy, 137 affected by resistant and intractable pain, and 22 affected by spastic rigidity were not managed, presenting a catastrophic evolution with most of them having a poor and not completely reversible functional outcome.

### 3.5. Results/Analysis of Neurosurgical Activity from 1 March 2021 up to 31 October 2021 (Alsace Region)

In this third period, where the availability of operating theaters increased from 2 to 3 a week in each hospital, the global neurosurgical activity regarding the case mix remained similar to the previous activity (Table 4). In this period, as in the previous one, neurosurgical functional procedures were totally and systematically cancelled, and due to a rebound of oncological diseases having the priority, all degenerative spine procedures were tremendously delayed, passing from a mean waiting list time of 35 days prior to the COVID-19 pandemic crisis to a delay of 150 days, resulting in huge medical, ethical, and social issues in terms of loss of functional outcome, quality of life, and burden of public finances. From 1 March 2021 to 31 October 2021, a total of 994 patients in the Alsace region affected by lumbar/cervical disc prolapse or cervical and dorso-lumbar degenerative stenosis were not surgically managed, leading the majority of them to have a suboptimal and/or poor and not reversible functional outcome.

### 3.6. Results/Analysis of Neurosurgical Activity from 1 November 2021 up to 15 March 2022 (Alsace Region)

In this fourth period, where the availability of operating theaters increased from 3 to 4 a week in each hospital, the global neurosurgical activity regarding the case mix changed moderately compared to the previous activity (Table 5). In this period, as in the previous one, neurosurgical functional procedures were still totally and systematically cancelled; however, all degenerative spine procedures for degenerative diseases started to progressively be treated under the standard of the previous pre-pandemic time. In terms of percentage, at the Strasbourg University Hospital, spine surgery represents about 35% of the annual activity, and it is almost double (70%) in the Colmar hospital. In this fourth period, the surgical spine activity progressively increased from 5 to 20% in Strasbourg and from 15 to 35% in Colmar. The latter was able to lower the surgery spine waiting list time from a mean 150-day delay to a mean of 135 days. Unfortunately, this 2-week decrease was still far from the standard prior to COVID-19. In this time frame, a total of 339 patients in the Alsace region affected by spinal diseases were not surgically treated. This represented an outstanding improvement in performance compared to the previous periods; however, the majority of the patients experienced a poorer and not reversible functional outcome.

## 4. Discussion

### 4.1. How COVID-19 Changed Daily Practices

In any crisis, a considerable level of adaptability, often referred to as “organizational agility,” is essential to navigate through dynamic challenges such as bed availability, staffing, equipment, supplies, and more. Our approach involved achieving a team consensus on finding the optimal balance between practical decisions and the ideal adherence to state-of-the-art practices. The primary objective was to deliver high-quality care without compromising standards through the strict implementation of military triage practices [31,32,33,34,35]. 

Externally, we capitalized on our national and international network, utilizing it to facilitate the evacuation of cases that posed a risk of overwhelming our capacity, redirecting them toward neighboring centers with more available ICU beds (including in Germany and Switzerland). As hospitals in the Grand Est (an administrative region including the former regions of Alsace, Champagne-Ardenne, and Lorraine) quickly became saturated with patients suffering from COVID-19, the health authorities deployed “Operation Resilience” to transfer hundreds of patients from hospitals under pressure to less congested hospitals, either by ambulance/helicopter/military plane or medically equipped bullet trains. 

The Army Health Service also, in conjunction, deployed a “mobile reanimation/ICU hospital” next to the civil hospital in Mulhouse (based on the hospital parking) between 21 March and 7 May 2020, creating 30 additional beds for patients suffering from respiratory insufficiency. Thanks to a medical military crew of 188, a total of 47 patients with a mean age of 60 years and an average inpatient stay of 15 days were treated.

In order to compare COVID-19 patients with respiratory distress and patients harboring neurosurgical lesions, three different criteria were evaluated, two of which were common in all the patients:

(a) The patient’s context: this criteria prioritized consideration for the patient’s own opinion whenever possible; the patient’s anticipated directive, if available; the involvement of the patient’s trusted person or family; and the patient’s age, clinical frailty score, prior modified Rankin score (mRS), Charlson comorbidity scale, and any recent worsening of cognitive status, autonomy, or comorbidities.
(b) The patient’s prognosis: this criteria was based on the global severity (simplified severity index score-2) and the specific severity (neurological for brain lesions, respiratory for COVID-19 patients). The prognostic criteria for brain-injured patients were specific to each brain injury itself: stroke, SAH, and brain tumor. In all cases, the clinical goal was to evaluate the patient’s chances of survival with a realistic probability of a mRS lower or equal to 3.

The investigation of this registry specific to the COVID-19 period has certainly helped in monitoring patients’ clinical trajectories and providing a more comprehensive understanding of the pandemic’s impact on surgical practices [36].

A decline was observed in high-speed head and spine traumas associated with nationwide restrictions on individual mobility. The traumatic lesions managed during lockdown consisted mostly of falls and domestic violence, as well as increased alcohol consumption [37,38]. Our emergency departments reported a 500% increase in alcohol-related injuries and hospitalizations. 

#### 4.1.1. Neurotrauma

Head and spinal injuries experienced only a relative reduction during the first wave of COVID-19, with their figures returning to pre-pandemic levels around the summer of 2020. Notably, the lockdown affected the mechanism of injury and reduced the incidence of many risk factors, including alcohol intoxication. This factor is not surprising: patterns of alcohol consumption changed during the pandemic, although those changes affected various strata of the population in different ways. Of note, alcohol consumption generally occurs in social settings, and in France and Belgium, the closure of bars and restaurants during the lockdown resulted in an overall reduction in alcohol consumption, especially among young adults. On the other hand, adult people between the ages of 35 and 50 reported that they drank more during the lockdown, even though they could not go to the aforementioned commercial services [39]. Additionally, ecological studies demonstrated that involuntary isolation during lockdown influenced alcohol-related preferences and reduced its overall consumption. For instance, a study conducted in Germany demonstrated that trait (predisposition) loneliness rather than state (momentary) loneliness was positively associated with alcohol consumption during the pandemic [40]. This might explain why toxicological tests for alcohol and drugs in drivers stopped at roadside checks increased not only during lockdown but also during the rest of the pandemic [41], and our findings of a significant rate of alcohol intoxication in many trauma patients align very well with such evidence.

#### 4.1.2. Brain Tumors

Brain tumors were the most common neurosurgical pathology. During the lockdown period, only 86 patients with a malignant brain lesion were managed either surgically or conservatively in the two hospitals. In the same period, 172 patients with brain cancer died before radiotherapy and chemotherapy protocols were reinstated in both hospitals. Furthermore, 104 patients died at home without any management as they were diagnosed either too late (when management was no longer reasonably indicated) or postmortem by autopsy. Globally, neurosurgical patients not undergoing oncology treatment (radio and chemotherapies) were 21%, compared to other specialties where this percentage of patients not receiving oncology treatment increased up to 65%. The consequences of the pandemic had a significant impact on the management of brain cancers [25,30,42,43,44,45,46,47,48]. Due to the need for social distancing and quarantine measures, many elective surgeries and non-urgent appointments were delayed or cancelled, leading to disruptions in the treatment of brain cancer patients. Additionally, the closure of many hospitals and clinics limited access to radiation therapy and chemotherapy. As a result, many brain cancer patients experienced delays in their treatment [49], and the latter situation was only minimally managed by the use of telemedicine. The pandemic has highlighted the importance of telemedicine and virtual consultations in the management of brain cancer patients, which should continue to be used and developed. 

Furthermore, many clinical trials in brain cancer were paused or delayed. It is difficult to provide an exact percentage impact of COVID-19 on the management of brain cancer data as it varies depending on the specific location and healthcare system. However, some studies have reported the following impacts: the number of brain cancer patients receiving surgery decreased by 30% during the initial peak of the pandemic in the United Kingdom in 2020 [50], the number of brain cancer patients starting radiation therapy decreased by 50% during the pandemic in the United States [51], and the number of brain cancer patients starting chemotherapy decreased by 70% during the pandemic in Italy [30]. These decreases were likely due to the temporary suspension of elective surgeries and treatments in order to prioritize COVID-19 patients and reduce the spread of the virus. Many healthcare systems eventually resumed regular surgical activity and implemented measures to safely treat brain cancer patients during the pandemic. In contrast, Rubens et al. [52] reported that COVID-19 was not associated with Clavien-Dindo grade IV complications, in-hospital mortality, or prolonged length of stay in the resection of intracranial meningioma in California. 

#### 4.1.3. Spine Disease

During lockdown, only 61 patients with spine disease were managed in our two hospitals: 51 surgically and 10 medically. The patients undergoing surgery presented abrupt and/or progressive neurological deficits with a high risk of permanent loss of different neurological functions. Elective spine surgery was completely cancelled during this period and restricted until 31 October 2021. This led to a large backlog of patients waiting for neurosurgeries, putting enormous strain on departments to resume their activities [53]. 

#### 4.1.4. Vascular Lesions 

The only neurosurgical subspecialty that was relatively spared was vascular [54,55]. Although all elective surgical and endovascular procedures for brain aneurysms and AVM were delayed, this did not affect their functional outcome. On the other hand, all emergent vascular cases were promptly managed either surgically or endovascularly. It is of note that during the lockdown period, the SAH following brain aneurysm bleeding decreased compared to the same period in 2019 [56], possibly due to national mobility restrictions reducing route fatigue and stress or reflecting unknown or misdiagnosed vascular cases. 

#### 4.1.5. Parkinson’s Disease, Dystonia (DBS), and Epilepsy 

One of the most affected subspecialties of neurosurgery was functional neurosurgery, especially in patients with Parkinson’s, dystonia, and drug-resistant epilepsy. In fact, all surgical functional procedures were cancelled during the two years, causing a significant loss of chance for functional recovery. Patients with Parkinson’s and dystonic diseases should receive operations in the so-called “fluctuating phase” (about 10 years after the beginning of the disease) and no later than 70 years of age to gain a theoretical 10–15 year benefit from DBS. Considering this information, all patients who were scheduled for surgery from March 2020 to March 2022 will not receive an operation because they are now older than 70 or beyond the fluctuating phase.

Similarly, for all the patients suffering from intractable and drug-resistant epilepsy, usually such patients are operated on as soon as the disease becomes resistant to medications, so all the patients scheduled for surgery from March 2020 to March 2022 that did not undergo surgery lost an important chance to treat the disease. Going forward, there is less of a chance of the patient responding positively to surgery and more time in which the patient’s personality disorganization has been negatively affected by epilepsy. In summary, if all such patients undergo surgery within the recommended therapeutic window, they can greatly benefit from the procedure; otherwise, after such a delay, the role of surgery is inconsistent and no longer recommended.

Due to the crisis, 90 patients with Parkinson’s disease and 53 with intractable and drug-resistant epilepsy in our region were not at all treated, causing a serious loss of chance for future outcomes and an increased financial burden for the social security system. Furthermore, regarding the other functional neurosurgery procedure, 137 patients affected by resistant and intractable pain and 22 affected by spastic rigidity were also not managed, causing a remarkable decrease in their functional recovery and various social and medical legal issues.

#### 4.1.6. Surgical Training

Many studies have investigated the impact of the COVID-19 pandemic on neurosurgical training; however, the drop in case volumes was not statistically significant enough to directly affect trainees’ learning curve. This aspect seems to be in keeping with the findings from our study: the task shifting and task sharing involved neurosurgical trainees and gave them an opportunity to acquire new valuable skills. Additionally, the recovery projects, for instance, those enabling a rebound of neuro-oncology procedures, allowed the trainees to maintain the operative numbers needed for their logbooks and appraisal of surgical competencies. Therefore, the progressive decline in neurosurgical trainees’ operative experience and surgical exposure compared to one decade ago, an issue that has been at the center of an ongoing debate in many European countries, might represent a multifactorial effect without a single identifiable cause [57,58]. 

Although an analysis of training during the pandemic goes beyond the scope of this study, it is worth mentioning that COVID-19 allowed not only the implementation of several measures for telemedicine but gave rise to a plethora of opportunities for virtual training through webinars, e-learning, and teleconferences. Many of those changes are still successful today. 

### 4.2. Insights Gained from the COVID-19 Pandemic in Neurosurgery Departments

With the rise of virtual consultations, it has become clear that telemedicine is a valuable tool for neurosurgeons. It helped reduce the spread of infection and also made it easier for patients who were unable to come to the hospital for an in-person outpatient clinic. The implementation of new tools, protocols, and ways of acting implies the following [59,60,61]:

(a) The need for flexible and adaptable treatment plans.
(b) The importance of infection control protocols, including the use of personal protective equipment, frequent cleaning of equipment and surfaces, and the need to isolate patients who are suspected or confirmed to have COVID-19.
(c) The importance of mental health support. The pandemic caused stress and anxiety for many patients, and this is particularly true for those who were dealing with serious health conditions. Neurosurgeons had to find ways to support the mental health of their patients, including providing counseling and support services.
(d) The need for collaboration and communication. The pandemic highlighted the importance of effective communication and collaboration among healthcare professionals. This includes working closely with other departments and specialists to provide the best possible care for patients, as well as sharing information and resources to help manage patients [14,62,63,64,65,66,67,68,69,70,71,72,73,74,75,76,77,78].

The long-term side effects of the COVID-19 pandemic on neurosurgical practice and diseases are still being studied and understood. A study published in the Journal of Neurosurgery also reported that the COVID-19 pandemic has led to a significant decrease in the number of neurosurgical procedures being performed, which could lead to a backlog of patients with untreated neurological conditions and an increased risk of long-term disability [1,2,14,79].

In order to quickly reduce the operating waiting list created by the COVID-19 crisis, in coordination with the ARS (Regional Health Authority), we created an important partnership with private hospitals to find additional operating slots. This new organization allowed surgeons, scrub nurses, and anesthetists to practice freely in public and private hospitals and, in only 9 months, lowered the number of patients waiting for elective neurosurgery by about 50%. We estimate that by September 2023, the entire backlog of patients might be resolved, and our system will start to run similarly to before the pandemic.

Some studies have reported on the potential long-term effects of the pandemic on neurological pathologies [80]. For example, commenting on the impact of COVID-19, The Lancet Neurology [81] highlighted that patients who had been hospitalized for COVID-19 infection had a higher risk of developing neurological complications such as ischemic and hemorrhagic stroke, encephalitis, and Guillain-Barre syndrome. Additionally, the study found that patients who had been hospitalized for COVID-19 were also more likely to experience long-term neurological problems such as fatigue, anxiety, and depression.

### 4.3. Future Challenges

The outlined organizational framework is viable for a limited duration and is applicable to any sudden healthcare crisis, though it would be unable to support care for a much longer period. This study confirmed that neurosurgeons must maintain flexibility in their practice and remain ready for adjustments to the provision of emergency neurosurgical care. Overall, the crucial factor for success lies in fostering close collaboration among specialties, both at the local level and, if required, extending to regional, national, and international levels, with well-structured coordination [82,83].

Moreover, the precise and ongoing documentation of unfolding events (war foci, mass migrations, etc.) and the consideration for scientific publications ideally reporting data from duly designed registries (like the one established in Alsace and described in this study), or at least through audits and morbidity and mortality meetings, will function as an observatory for future generations of neurosurgeons.

As we now live in a post-pandemic stage, it is imperative not to overlook the profound psychological, social, and financial consequences that might exist in the near future. The pandemic has resulted in a significant reduction in financial income and an increase in expenses for many hospitals and clinics. Neurosurgery departments must manage financial challenges by reducing costs, increasing efficiency, and finding new sources of funding [84].

From a global point of view, governments and organizations should bear in mind all they learned from the COVID-19 crisis and improve their preparedness for future pandemics by studying, expanding, and implementing emergency strategies and plans through investment in healthcare infrastructure, strengthening global health systems, and enhancing international cooperation. The pandemic has also exposed and exacerbated existing social and political divisions. Rebuilding trust in institutions and addressing these divisions represents one of the most important and significant challenges to manage.

## 5. Conclusions

COVID-19 has fostered collaboration within our clinical and managerial workforce, fostering discussions, idea-sharing, and concerted efforts to optimize all available resources in dealing with this devastating crisis. We believe that we extracted the best from each other, enabling us to leverage this experience within a few months and restart routine elective practices [78].

This study illustrates that the swift reorganization of neurosurgical care in the Alsace region, meticulously implemented in the early stages of the initial wave of the COVID-19 pandemic, facilitated the safe, timely, and effective performance of a diverse range of surgical procedures. Despite a significant drop in surgical volume disrupting routine practice, the documented case mix suggests that through judicious organizational planning, clinical triaging, and prioritization, we successfully managed pathologies across the entire neurosurgical spectrum despite the constraints imposed by the healthcare crisis. Simultaneously, we organized and coordinated the immediate and mid-term post-COVID-19 phases.

## Figures and Tables

**Table 1:** Classification of neurosurgical interventions according to degree of emergency. (Reproduced from [3]).

Class	Type of Intervention	Management
Emergency (EM group)	Head/spine trauma, intracranial bleeding (due to ruptured vascular malformation), acute hydrocephalus, and head/spine oncologic cases with rapid onset of medically refractory intracranial hypertension or risk of permanent neurological deficit.	No need for a swab prior to surgical intervention
Deferrable (UP group)	Head/spine oncology cases showing a slowly progressive neurological deficit responding to steroid therapy (e.g., high-grade glioma, brain metastasis, meningioma, pituitary adenoma, etc.). Large disc herniation with impending cauda equine syndrome.	Management after swab, surgical intervention deferrable at least 48 h, and expedited within 7–15 days
Elective (EL group)	Any benign tumor or other pathology not causing an irreversible neurological deficit or putting patients in jeopardy.	Management after swab, surgical intervention re-scheduled within 2–4 months

**Table 2:** Neurosurgical care during the lockdown by pathologies.

Pathologies	Surgical/Interventional Cases [EM/UP/EL]	Non-Surgical Cases	Total
**Trauma**	**47 [12/31/4]**	**50**	**97 (29.8%)**
Chronic subdural hematoma	30 [6/20/4]	–	30
Head trauma alone	8 [6/2/–]	31	39
Craniovertebral trauma	3 [1/2/–]	4	7
Spine trauma alone	6 [1/5/–]	15	21
**Neuro-oncology**	**52 [18/25/9]**	**34**	**86 (26.5%)**
Metastasis	20 [10/10/–]	16	36
Glioblastoma	16 [3/7/6]	8	24
Meningioma	4 [1/3/–]	8	12
Lymphoma	2 [–/2/–]	–	2
Ependymoma	3 [–/1/2]	–	3
Craniopharyngioma	1 [–/–/1]	–	1
Pituitary adenoma	3 [3/–/–]	2	5
Ethmoidal adenocarcinoma	1 [1/–/–]	–	1
PNET	2 [–/2/–]	–	2
**Degenerative spinal disease**	**51 [6/28/17]**	**10**	**61 (18.8%)**
Degenerartive Cervical Myelopathy	14 [–/5/9]	–	14
Cervico-brachial neuralgia	2 [–/–/2]	2	2
Hyperalgic sciatalgia	25 [5/16/4]	8	33
Herniated lumbar disc with motor deficit	3 [1/2/–]	–	3
Lumbar stenosis	7 [–/5/2]	–	7
**Neurovascular**	**13 [9/4/0]**	**17**	**30 (9.2%)**
Aneurismal subarachnoid hemorrhage	6 [6/–/–]	1	7
Non-aneurismal subarachnoid hemorrhage	1 [1/–/–]	7	8
Spontaneous intracerebral hemorrhage	3 [1/2/–]	2	5
Cavernoma	2 [–/2/–]	3	5
Malignant sylvian ischemic stroke	1 [1/–/–]	2	3
Spontaneous epidural cervical hematoma	–	2	2
**Infection**	**17 [9/5/3]**	**6**	**23 (7.1%)**
Foreign material infections	10 [3/4/3]	–	10
Cerebral abscess	3 [3/–/–]	–	3
Cerebral opportunist infection	1 [–/1/–]	3	4
Meningo-encephalitis	–	2	2
Scar reopening	–	1	1
Infectious discitis with epidural collection	3 [3/–/–]	–	3
**Functional**	**0 [–/–/–]**	**18**	**18 (5.5%)**
**Hydrocephalus (acute shunt dysfunction)**	**9 [6/3/0]**	**1**	**10 (3.1%)**
**Neurosurgical care**	**189 (58.2%) [60/96/33]**	**136 (41.8%)**	**325**

The degrees of emergency for requiring surgical treatment were classified as immediate (within hours) for EM (emergency), within a maximum of 7–15 days for UP (deferrable), and within 2 months for EL (elective).

**Table 3:** Neurosurgical care during the period from 1 June 2020 until 28 February 2021 by pathologies.

Pathologies	Surgical/Interventional Cases [EM/UP/EL]	Non-Surgical Cases	Total
**Trauma**	**196 [74/88/34]**	**254**	**450 (39.0%)**
Chronic subdural hematoma	92 [30/48/14]	14	106
Head trauma alone	60 [24/24/12]	108	168
Craniovertebral trauma	12 [4/8/–]	44	56
Spine trauma alone	32 [16/8/8]	88	120
**Neuro-oncology**	**200 [38/107/55]**	**126**	**326 (28.3%)**
Metastasis	88 [18/60/10]	30	118
Glioblastoma	60 [16/32/12]	25	85
Meningioma	14 [-/4/10]	36	50
Lymphoma	10 [-/10/-]	–	10
Ependymoma	5 [–/1/4]	10	15
Craniopharyngioma	5 [–/–/5]	–	5
Pituitary adenoma	18 [4/-/14]	25	43
Ethmoidal adenocarcinoma	0 [–/–/–]	–	0
PNET	0 [–/–/–]	–	0
**Degenerative spinal disease**	**68 [6/20/42]**	**38**	**106 (9.2%)**
Degenerative Cervical Myelopathy	24 [–/10/14]	12	36
Cervico-brachial neuralgia	12 [2/–/10]	12	24
Hyperalgic sciatalgia	28 [4/8/16]	12	40
Herniated lumbar disc with motor deficit	2 [–/2/–]	–	2
Lumbar stenosis	2 [–/–/2]	2	4
**Neurovascular**	**93 [87/6/0]**	**62**	**155 (13.5%)**
Aneurismal subarachnoid hemorrhage	30 [30/–/–]	12	42
Non-aneurismal subarachnoid hemorrhage	10 [10/–/–]	30	40
Spontaneous intracerebral hemorrhage	25 [25/–/–]	4	29
Cavernoma	12 [8/4/–]	10	22
Malignant sylvian ischemic stroke	14 [14/–/–]	4	18
Spontaneous epidural cervical hematoma	2 [–/2/–]	2	4
**Infection**	**44 [32/8/4]**	**16**	**60 (5.2%)**
Foreign material infections	16 [4/8/4]	4	20
Cerebral abscess	22 [22/-/-]	–	22
Cerebral opportunist infection	4 [4/–/–]	–	4
Meningo-encephalitis	–	8	8
Scar reopening	–	4	4
Infectious discitis with epidural collection	2 [2/–/–]	–	2
**Functional**	**0 [–/–/–]**	**12**	**12 (1.0%)**
**Hydrocephalus (acute shunt dysfunction)**	**40 [28/12/0]**	**4**	**44 (3.8%)**
**Neurosurgical care**	**641 (55.6%) [265/241/135]**	**512 (44.4%)**	**1153**

The degrees of emergency for requiring surgical treatment were classified as immediate (within hours) for EM (emergency), within a maximum of 7–15 days for UP (deferrable), and within 2 months for EL (elective).

**Table 4:** Neurosurgical care during the period from 1 March 2021 until 31 October 2021 by pathologies.

Pathologies	Surgical/Interventional Cases [EM/UP/EL]	Non-Surgical Cases	Total
**Trauma**	**235 [116/104/15]**	**242**	**477 (34.2%)**
Chronic subdural hematoma	102 [30/68/4]	25	127
Head trauma alone	70 [44/18/8]	145	215
Craniovertebral trauma	18 [16/2/–]	19	37
Spine trauma alone	45 [26/16/3]	53	98
**Neuro-oncology**	**296 [39/178/79]**	**166**	**462 (33.1%)**
Metastasis	110 [14/84/12]	48	158
Glioblastoma	92 [20/62/10]	37	129
Meningioma	30 [–/6/24]	40	70
Lymphoma	14 [–/14/–]	–	14
Ependymoma	9 [–/7/2]	6	15
Craniopharyngioma	4 [–/–/4]	–	4
Pituitary adenoma	30 [5/–/25]	35	65
Ethmoidal adenocarcinoma	2 [–/–/2]	–	2
PNET	5 [–/5/–]	–	5
**Degenerative spinal disease**	**80 [9/30/41]**	**35**	**115 (8.3%)**
Degenerative Cervical Myelopathy	35 [–/18/17]	12	47
Cervico-brachial neuralgia	18 [4/–/14]	14	32
Hyperalgic sciatalgia	23 [5/10/8]	9	32
Herniated lumbar disc with motor deficit	2 [–/2/–]	–	2
Lumbar stenosis	2 [–/–/2]	–	2
**Neurovascular**	**143 [125/4/14]**	**55**	**198 (14.2%)**
Aneurismal subarachnoid hemorrhage	45 [45/–/–]	15	60
Non-aneurismal subarachnoid hemorrhage	15 [15/–/–]	8	23
Spontaneous intracerebral hemorrhage	35 [29/6/–]	7	42
Cavernoma	18 [–/4/14]	15	33
Malignant sylvian ischemic stroke	30 [30/–/–]	6	36
Spontaneous epidural cervical hematoma	0 [–/–/–]	4	4
**Infection**	**50 [36/12/2]**	**4**	**54 (3.9%)**
Foreign material infections	20 [6/12/2]	4	24
Cerebral abscess	18 [18/–/–]	–	18
Cerebral opportunist infection	12 [12/–/–]	–	12
Meningo-encephalitis	–	–	
Scar reopening	–	–	
Infectious discitis with epidural collection	–	–	
**Functional**	0 [–/–/–]	16	16 (1.1%)
**Hydrocephalus (acute shunt dysfunction)**	68 [48/20/0]	4	72 (5.2%)
**Neurosurgical care**	**872 (62.6%) [367/354/151]**	**522 (37.4%)**	**1394**

The degrees of emergency for requiring surgical treatment were classified as immediate (within hours) for EM (emergency), within a maximum of 7–15 days for UP (deferrable), and within 2 months for EL (elective).

**Table 5:** Neurosurgical care during the period from 1 November 2021 until 15 March 2022 by pathologies.

Pathologies	Surgical/Interventional Cases [EM/UP/EL]	Non-Surgical Cases	Total
**Trauma**	**190 [71/87/32]**	**140**	**330 (34.0%)**
Chronic subdural hematoma	65 [10/45/10]	15	80
Head trauma alone	70 [35/20/15]	65	135
Craniovertebral trauma	10 [6/2/2]	25	35
Spine trauma alone	45 [20/20/5]	35	80
**Neuro-oncology**	**250 [35/116/99]**	**80**	**330 (34.0%)**
Metastasis	75 [10/55/10]	25	100
Glioblastoma	55 [15/35/5]	15	70
Meningioma	20 [–/4/16]	25	45
Lymphoma	25 [–/20/5]	–	25
Ependymoma	7 [–/–/7]	–	7
Craniopharyngioma	8 [–/2/6]	–	8
Pituitary adenoma	60 [10/–/50]	15	75
Ethmoidal adenocarcinoma	–	–	–
PNET	–	–	–
**Degenerative spinal disease**	**50 [4/12/34]**	**25**	**75 (7.7%)**
Degenerative Cervical Myelopathy	18 [–/4/14]	8	26
Cervico-brachial neuralgia	12 [–/4/8]	6	18
Hyperalgic sciatalgia	12 [4/–/8]	6	18
Herniated lumbar disc with motor deficit	4 [–/4/–]	3	7
Lumbar stenosis	4 [–/–/4]	2	6
**Neurovascular**	**80 [70/2/8]**	**25**	**105 (10.8%)**
Aneurismal subarachnoid hemorrhage	25 [25/–/–]	5	30
Non-aneurismal subarachnoid hemorrhage	10 [10/–/–]	2	12
Spontaneous intracerebral hemorrhage	25 [25/–/–]	8	33
Cavernoma	8 [–//8]	4	12
Malignant sylvian ischemic stroke	10 [10/–/–]	2	12
Spontaneous epidural cervical hematoma	2 [–/2/–]	4	6
**Infection**	**40 [26/12/2]**	**10**	**50 (5.2%)**
Foreign material infections	10 [6/2/2]	2	12
Cerebral abscess	10 [10/–/–]	–	10
Cerebral opportunist infection	4 [4/–/–]	–	4
Meningo-encephalitis	6 [6/–/–]	–	6
Scar reopening	6 [–/6/–]	4	10
Infectious discitis with epidural collection	4 [–/4/–]	4	8
**Functional**	**0 [–/–/–]**	**30**	**30 (3.1%)**
**Hydrocephalus (acute shunt dysfunction)**	**40 [25/12/3]**	**10**	**50 (5.2%)**
**Neurosurgical care**	**650 (67.0%) [231/241/178]**	**320 (33.0%)**	**970**

The degrees of emergency for requiring surgical treatment were classified as immediate (within hours) for EM (emergency), within a maximum of 7–15 days for UP (deferrable), and within 2 months for EL (elective).

## Data Availability

Data are contained within the article.

## References

[1] Ashkan K., Jung J., Velicu A.M., Raslan A., Faruque M., Kulkarni P., Bleil C., Hasegawa H., Kailaya-Vasan A., Maratos E. (2021). Neurosurgery and Coronavirus: Impact and Challenges—Lessons Learnt from the First Wave of a Global Pandemic. Acta Neurochir..

[2] Bajunaid K., Alqurashi A., Alatar A., Alkutbi M., Alzahrani A.H., Sabbagh A.J., Alobaid A., Barnawi A., Alferayan A.A., Alkhani A.M. (2020). Neurosurgical Procedures and Safety during the COVID-19 Pandemic: A Case-Control Multicenter Study. World Neurosurg..

[3] Chibbaro S., Ganau M., Todeschi J., Proust F., Cebula H. (2020). How SARS-CoV-2 Is Forcing Us to Reconsider and Reorganize Our Daily Neurosurgical Practice. Neurochirurgie.

[4] COVID—Coronavirus Statistics—Worldometer. https://www.worldometers.info/coronavirus/.

[5] Balak N., Inan D., Ganau M., Zoia C., Sönmez S., Kurt B., Akgül A., Tez M. (2021). A Simple Mathematical Tool to Forecast COVID-19 Cumulative Case Numbers. Clin. Epidemiol. Glob. Health.

[6] Burke J.F., Chan A.K., Mummaneni V., Chou D., Lobo E.P., Berger M.S., Theodosopoulos P.V., Mummaneni P.V. (2020). Letter: The Coronavirus Disease 2019 Global Pandemic: A Neurosurgical Treatment Algorithm. Neurosurgery.

[7] Nepogodiev D., Simoes J.F., Li E., Picciochi M., Glasbey J.C., Baiocchi G., Blanco-Colino R., Chaudhry D., COVIDSurg Collaborative, GlobalSurg Collaborative (2021). Effects of Pre-operative Isolation on Postoperative Pulmonary Complications after Elective Surgery: An International Prospective Cohort Study. Anaesthesia.

[8] COVIDSurg Collaborative (2020). Elective Surgery Cancellations Due to the COVID-19 Pandemic: Global Predictive Modelling to Inform Surgical Recovery Plans. Br. J. Surg..

[9] Finn R., Ganau M., Jenkinson M.D., Plaha P. (2021). COVID-Legal Study: Neurosurgeon Experience in Britain during the First Phase of the COVID-19 Pandemic—Medico-Legal Considerations. Br. J. Neurosurg..

[10] Fontanella M.M., De Maria L., Zanin L., Saraceno G., Terzi Di Bergamo L., Servadei F., Chaurasia B., Olivi A., Vajkoczy P., Schaller K. (2020). Neurosurgical Practice during the Severe Acute Respiratory Syndrome Coronavirus 2 (SARS-CoV-2) Pandemic: A Worldwide Survey. World Neurosurg..

[11] Glasbey J.C., Nepogodiev D., Simoes J.F.F., Omar O., Li E., Venn M.L., Abou Chaar M.K., Capizzi V., Chaudhry D., Desai A. (2021). Elective Cancer Surgery in COVID-19–Free Surgical Pathways during the SARS-CoV-2 Pandemic: An International, Multicenter, Comparative Cohort Study. JCO.

[12] COVIDSurg Collaborative (2022). Projecting COVID-19 Disruption to Elective Surgery. Lancet.

[13] COVID-19 National Preparedness Collaborators (2022). Pandemic Preparedness and COVID-19: An Exploratory Analysis of Infection and Fatality Rates, and Contextual Factors Associated with Preparedness in 177 Countries, from Jan 1, 2020, to Sept 30, 2021. Lancet.

[14] Nepogodiev D., Simoes J.F., Li E., Picciochi M., Glasbey J.C., Baiocchi G., Blanco-Colino R., Chaudhry D., COVIDSurg Collaborative, GlobalSurg Collaborative (2022). SARS-CoV-2 Infection and Venous Thromboembolism after Surgery: An International Prospective Cohort Study. Anaesthesia.

[15] COVIDSurg Collaborative (2020). Mortality and Pulmonary Complications in Patients Undergoing Surgery with Perioperative SARS-CoV-2 Infection: An International Cohort Study. Lancet.

[16] Coronado-Gutiérrez D., Ganau S., Bargalló X., Úbeda B., Porta M., Sanfeliu E., Burgos-Artizzu X.P. (2022). Quantitative Ultrasound Image Analysis of Axillary Lymph Nodes to Differentiate Malignancy from Reactive Benign Changes due to COVID-19 Vaccination. Eur. J. Radiol..

[17] Flexman A.M., Abcejo A.S., Avitsian R., De Sloovere V., Highton D., Juul N., Li S., Meng L., Paisansathan C., Rath G.P. (2020). Neuroanesthesia Practice during the COVID-19 Pandemic: Recommendations from Society for Neuroscience in Anesthesiology and Critical Care (SNACC). J. Neurosurg. Anesthesiol..

[18] Germanò A., Raffa G., Angileri F.F., Cardali S.M., Tomasello F. (2020). Coronavirus Disease 2019 (COVID-19) and Neurosurgery: Literature and Neurosurgical Societies Recommendations Update. World Neurosurg..

[19] Grelat M., Pommier B., Portet S., Amelot A., Barrey C., Leroy H.-A., Madkouri R. (2020). Patients with Coronavirus 2019 (COVID-19) and Surgery: Guidelines and Checklist Proposal. World Neurosurg..

[20] Muhammad S., Tanikawa R., Lawton M.T., Niemelä M., Hänggi D. (2020). Letter: Safety Instructions for Neurosurgeons during COVID-19 Pandemic Based on Recent Knowledge and Experience. Neurosurgery.

[21] Tan Y., Wang J., Zhao K., Han L., Zhang H., Niu H., Shu K., Lei T. (2020). Preliminary Recommendations for Surgical Practice of Neurosurgery Department in the Central Epidemic Area of 2019 Coronavirus Infection. Curr. Med. Sci..

[22] Ganau M., Netuka D., Broekman M., Zoia C., Tsianaka E., Schwake M., Balak N., Sekhar A., Ridwan S., Clusmann H. (2020). Neurosurgeons and the Fight with COVID-19: A Position Statement from the EANS Individual Membership Committee. Acta Neurochir..

[23] Dannhoff G., Cebula H., Chibbaro S., Ganau M., Todeschi J., Mallereau C.-H., Pottecher J., Proust F., Ollivier I. (2021). Investigating the Real Impact of COVID-19 Pandemic on the Daily Neurosurgical Practice?. Neurochirurgie.

[24] Chaudhry R., Dranitsaris G., Mubashir T., Bartoszko J., Riazi S. (2020). A Country Level Analysis Measuring the Impact of Government Actions, Country Preparedness and Socioeconomic Factors on COVID-19 Mortality and Related Health Outcomes. EClinicalMedicine.

[25] Hu Y.-J., Zhang J., Chen Z. (2020). Experiences of Practicing Surgical Neuro-Oncology during the COVID-19 Pandemic. J. Neuro-Oncol..

[26] Quaquarini E., Saltalamacchia G., Presti D., Caldana G., Tibollo V., Malovini A., Palumbo R., Teragni C.M., Balletti E., Mollica L. (2020). Impact of COVID-19 Outbreak on Cancer Patient Care and Treatment: Data from an Outpatient Oncology Clinic in Lombardy (Italy). Cancers.

[27] Wen J., Qi X., Lyon K.A., Liang B., Wang X., Feng D., Huang J.H. (2020). Lessons from China When Performing Neurosurgical Procedures during the Coronavirus Disease 2019 (COVID-19) Pandemic. World Neurosurg..

[28] Zoia C., Bongetta D., Veiceschi P., Cenzato M., Di Meco F., Locatelli D., Boeris D., Fontanella M.M. (2020). Neurosurgery during the COVID-19 Pandemic: Update from Lombardy, Northern Italy. Acta Neurochir..

[29] Poorolajal J., Cheraghi Z., Irani A.D., Rezaeian S. (2011). Quality of Cohort Studies Reporting Post the Strengthening the Reporting of Observational Studies in Epidemiology (STROBE) Statement. Epidemiol. Health.

[30] Ramakrishna R., Zadeh G., Sheehan J.P., Aghi M.K. (2020). Inpatient and Outpatient Case Prioritization for Patients with Neuro-Oncologic Disease amid the COVID-19 Pandemic: General Guidance for Neuro-Oncology Practitioners from the AANS/CNS Tumor Section and Society for Neuro-Oncology. J. Neuro-Oncol..

[31] Jean W.C., Ironside N.T., Sack K.D., Felbaum D.R., Syed H.R. (2020). The Impact of COVID-19 on Neurosurgeons and the Strategy for Triaging Non-Emergent Operations: A Global Neurosurgery Study. Acta Neurochir..

[32] Lucas T. (2020). Letter: Neurosurgical Triage in the Pandemic Era. Neurosurgery.

[33] Pesce A., Palmieri M., Armocida D., Frati A., Santoro A. (2020). Neurosurgery and Coronavirus (COVID-19) Epidemic: Doing Our Part. Neurosurgery.

[34] Rubulotta F., Soliman-Aboumarie H., Filbey K., Geldner G., Kuck K., Ganau M., Hemmerling T.M. (2020). Technologies to Optimize the Care of Severe COVID-19 Patients for Health Care Providers Challenged by Limited Resources. Anesth. Analg..

[35] Schaller K. (2020). Neurosurgeons in the Corona Crisis: Striving for Remedy and Redemption. A Message from the President of the EANS. Acta Neurochir..

[36] Zaed I., Servadei F. (2022). The Importance of Clinical Registries in Neurosurgery: Is It Time for a European Registry?. J. Neurosurg..

[37] Abdo C., Miranda E., Santos C., Júnior J.B., Bernardo W. (2020). Domestic Violence and Substance Abuse during COVID19: A Systematic Review. Indian J. Psychiatry.

[38] Chikritzhs T., Livingston M. (2021). Alcohol and the Risk of Injury. Nutrients.

[39] Pabst A., Bollen Z., Creupelandt C., Fontesse S., Maurage P. (2021). Alcohol Consumption Changes following COVID-19 Lockdown among French-Speaking Belgian Individuals at Risk for Alcohol Use Disorder. Prog. Neuro-Psychopharmacol. Biol. Psychiatry.

[40] Haucke M., Heinzel S., Liu S. (2024). Involuntary Social Isolation and Alcohol Consumption: An Ecological Momentary Assessment in Germany amid the COVID-19 Pandemic. Alcohol Alcohol..

[41] Marrone M., Pititto F., Stellacci A., Nicolì S., Buongiorno L., De Luca B.P., Aventaggiato L., Strisciullo G., Solarino B., Benevento M. (2023). Alcohol and Drug Consumption among Drivers before and during the COVID-19 Pandemic: An Observational Study. EJIHPE.

[42] Bernhardt D., Wick W., Weiss S.E., Sahgal A., Lo S.S., Suh J.H., Chang E.L., Foote M., Perry J., Meyer B. (2020). Neuro-Oncology Management during the COVID-19 Pandemic with a Focus on WHO Grades III and IV Gliomas. Neuro-Oncology.

[43] COVIDSurg Collaborative (2021). Effect of COVID-19 Pandemic Lockdowns on Planned Cancer Surgery for 15 Tumour Types in 61 Countries: An International, Prospective, Cohort Study. Lancet Oncol..

[44] Ganau L., Ligarotti G.K.I., Ganau M. (2018). Predicting Complexity of Tumor Removal and Postoperative Outcome in Patients with High-Grade Gliomas. Neurosurg. Rev..

[45] Hanna T.P., King W.D., Thibodeau S., Jalink M., Paulin G.A., Harvey-Jones E., O’Sullivan D.E., Booth C.M., Sullivan R., Aggarwal A. (2020). Mortality Due to Cancer Treatment Delay: Systematic Review and Meta-Analysis. BMJ.

[46] Lai A.G., Pasea L., Banerjee A., Hall G., Denaxas S., Chang W.H., Katsoulis M., Williams B., Pillay D., Noursadeghi M. (2020). Estimated Impact of the COVID-19 Pandemic on Cancer Services and Excess 1-Year Mortality in People with Cancer and Multimorbidity: Near Real-Time Data on Cancer Care, Cancer Deaths and a Population-Based Cohort Study. BMJ Open.

[47] Mohile N.A., Blakeley J.O., Gatson N.T.N., Hottinger A.F., Lassman A.B., Ney D.E., Olar A., Schiff D., Shih H.A., Strowd R. (2020). Urgent Considerations for the Neuro-Oncologic Treatment of Patients with Gliomas During the COVID-19 Pandemic. Neuro-Oncology.

[48] GlobalSurg Collaborative and National Institute for Health Research Global Health Research Unit on Global Surgery (2021). Global Variation in Postoperative Mortality and Complications after Cancer Surgery: A Multicentre, Prospective Cohort Study in 82 Countries. Lancet.

[49] COVIDSurg Collaborative (2020). Delaying Surgery for Patients with a Previous SARS-CoV-2 Infection. Br. J. Surg..

[50] Mitchell H., Alford B.S., O’Hare S., O’Callaghan E., Fox C., Gavin A.T. (2022). Impact of the COVID-19 Pandemic on Emergency Hospital Cancer Admissions in a UK Region. BMC Cancer.

[51] Mireștean C.C., Agop M., Buzea C.G., Cazacu M.M., Prelipceanu M., Iancu R.I., Iancu D.T. (2021). Radiotherapy Challenges in COVID Era. Biomedical Engineering Tools for Management for Patients with COVID-19.

[52] Rubens M., Saxena A., Ramamoorthy V., Ahmed M.A., Zhang Z., McGranaghan P., Veledar E., McDermott M. (2022). Impact of COVID-19 on Intracranial Meningioma Resection: Results from California State Inpatient Database. Cancers.

[53] Minhas Z., Ganau M., Thakar C., Reynolds J., Rothenfluh D., Bojanic S., Grannum S., Chaudhary B.R., Pyrovolou N., Sikander M. (2020). COVID-19: New Challenges, Risks, and the Future Provision of Care in Spinal Services. Bone Jt. J..

[54] Nguyen T.N., Qureshi M.M., Klein P., Yamagami H., Mikulik R., Czlonkowska A., Abdalkader M., Sedova P., Sathya A., Lo H.C. (2023). Global Impact of the COVID-19 Pandemic on Stroke Volumes and Cerebrovascular Events: A 1-Year Follow-Up. Neurology.

[55] Strambo D., Marto J.P., Ntaios G., Nguyen T.N., Michel P., Herzig R., Członkowksa A., Demeestere J., Yassin Mansour O., Georgiopoulos G. (2024). Effect of Asymptomatic and Symptomatic COVID-19 on Acute Ischemic Stroke Revascularization Outcomes. Stroke.

[56] Siegler J.E., Heslin M.E., Thau L., Smith A., Jovin T.G. (2020). Falling Stroke Rates during COVID-19 Pandemic at a Comprehensive Stroke Center. J. Stroke Cerebrovasc. Dis..

[57] Khan A., Mao J.Z., Soliman M.A.R., Rho K., Hess R.M., Reynolds R.M., Riley J.P., Mullin J.P., Siddiqui A.H., Levy E.I. (2021). The Effect of COVID-19 on Trainee Operative Experience at a Multihospital Academic Neurosurgical Practice: A First Look at Case Numbers. Surg. Neurol. Int..

[58] Alimohammadi E., Eden S.V., Anand S.K., Ahadi P., Bostani A., Bagheri S.R. (2022). The Impact of Coronavirus 2019 (COVID-19) on Neurosurgical Practice and Training: A Review Article. Br. J. Neurosurg..

[59] Blue R., Yang A.I., Zhou C., De Ravin E., Teng C.W., Arguelles G.R., Huang V., Wathen C., Miranda S.P., Marcotte P. (2020). Telemedicine in the Era of Coronavirus Disease 2019 (COVID-19): A Neurosurgical Perspective. World Neurosurg..

[60] Schaller K. (2016). Editorial Re: WhatsAPP in Neurosurgery: The Best Practice Is in Our Hands. Acta Neurochir..

[61] Ameel M., Myllynen M., Kallakorpi S. (2022). Exploring Hybrid Leadership: Experiences of Remote Leadership in Healthcare. JONA J. Nurs. Adm..

[62] Chang T.F.H., Baelen R.N., Ramburn T.T., Purandare P. (2022). Developing Positive Self-Leadership through “Inner Engineering”. JMD.

[63] Van Dongen L.J.C., Hafsteinsdóttir T.B. (2022). Leadership of PhD-prepared Nurses Working in Hospitals and Its Influence on Career Development: A Qualitative Study. J. Clin. Nurs..

[64] Ecoff L., Stichler J.F. (2022). Development and Psychometric Testing of a Leadership Competency Assessment. JONA J. Nurs. Adm..

[65] Haddad M.E., Faithfull-Byrne A., Thompson L., Wilkinson G., Moss C. (2022). Nurse Unit Managers’ Work and Impacts on Clinical Leadership: A Cross-Sectional Study. Collegian.

[66] Harmon J., Howard M., Sharrad S. (2022). Habitus, Social Capital, Leadership, and Reflection: Insights for Early Career Nurse Academics. Collegian.

[67] Iorio-Morin C., Hodaie M., Sarica C., Dea N., Westwick H.J., Christie S.D., McDonald P.J., Labidi M., Farmer J.-P., Brisebois S. (2020). Letter: The Risk of COVID-19 Infection during Neurosurgical Procedures: A Review of Severe Acute Respiratory Distress Syndrome Coronavirus 2 (SARS-CoV-2) Modes of Transmission and Proposed Neurosurgery-Specific Measures for Mitigation. Neurosurgery.

[68] Jain A., Brown G., Hudson H.T., Patel A., Herrera F.A. (2022). A Leadership Perspective on the Plastic and Reconstructive Surgery Residency Application Cycle during the COVID-19 Pandemic. JPRAS Open.

[69] Lewis C.P., Aldossari M. (2022). “*One of These Things Is Not like the Others*”: The Role of Authentic Leadership in Cross-Cultural Leadership Development. LODJ.

[70] Malak H., Mirza B., Rundio A., Mirza M. (2022). Impact of Practicing Servant Leadership Style among Chief Nursing Officers (CNOs) in Nursing Organizations. J. Interprof. Educ. Pract..

[71] Morris-Wiseman L.F., Dent D., Nfonsam V.N., Arora T.K. (2022). Leadership Diversity in the Association of Program Directors in Surgery: A Report of Progress. J. Surg. Educ..

[72] Nikpour J., Hickman R.L., Clayton-Jones D., Gonzalez-Guarda R.M., Broome M.E. (2022). Inclusive Leadership to Guide Nursing’s Response to Improving Health Equity. Nurs. Outlook.

[73] Singh A., Spadaro K. (2022). Leadership Self and Means Efficacy among Nursing Faculty: A National Study. J. Nurs. Educ..

[74] Solbakken R., Bondas T., Kasén A. (2022). Relationships Influencing Caring in First-line Nursing Leadership: A Visual Hermeneutic Study. Scand. Caring Sci..

[75] McLean K.A., Kamarajah S.K., Chaudhry D., Gujjuri R.R., Raubenheimer K., Trout I., Al Ameer E., Creagh-Brown B., Harrison E.M., STARSurg Collaborative and COVIDSurg Collaborative (2021). Death following Pulmonary Complications of Surgery before and during the SARS-CoV-2 Pandemic. Br. J. Surg..

[76] Williams T., Hande K., Kleinpell R. (2022). Linking Process Improvement with DNP Projects: Strategies to Advance Clinical Leadership Initiatives. Nurse Lead..

[77] Yau A.A., Cortez P., Auguste B.L. (2022). The Physician Leader: Teaching Leadership in Medicine. Adv. Chronic Kidney Dis..

[78] Ganau M., Foroni R.I., Gerosa M., Ricciardi G.K., Longhi M., Nicolato A. (2015). Radiosurgical Options in Neuro-Oncology: A Review on Current Tenets and Future Opportunities. Part II: Adjuvant Radiobiological Tools. Tumori.

[79] Arraya M. (2022). The Relationship between Distinctive Capabilities System, Learning Orientation, Leadership and Performance. EJMS.

[80] Tartara F., Cofano F., Zenga F., Boeris D., Garbossa D., Cenzato M. (2020). Are We Forgetting Non-COVID-19-Related Diseases during Lockdown?. Acta Neurochir..

[81] The Lancet Neurology (2020). The Neurological Impact of COVID-19. Lancet Neurol..

[82] Tomasi S.O., Umana G.E., Raudino G., Scalia G., Ganau M., Winkler P.A. (2020). In Reply: Neurosurgery and Coronavirus (COVID-19) Epidemic: Doing Our Part. Neurosurg. Open.

[83] Torti J.M.I., Inayat H., Inayat A., Lingard L., Haddara W., Sultan N. (2022). Perspectives on Physician Leadership: The Role of Character-based Leadership in Medicine. Med. Educ..

[84] Sivakanthan S., Pan J., Kim L., Ellenbogen R., Saigal R. (2020). Economic Impact of COVID-19 on a High-Volume Academic Neurosurgical Practice. World Neurosurg..

